# Among people who use heroin, tobacco smoking and illegal drugs cause a similar number of premature deaths

**DOI:** 10.1111/add.70140

**Published:** 2025-07-17

**Authors:** Dan Lewer, Harry Tattan‐Birch, Sharon Cox

**Affiliations:** ^1^ Department of Epidemiology and Public Health University College London London UK; ^2^ Bradford Centre for Health Data Science Bradford Institute for Health Research Bradford UK; ^3^ Department of Behavioural Science and Health University College London London UK

**Keywords:** demography, heroin, illegal drugs, life expectancy, life tables, mortality, smoking, tobacco smoking

## Abstract

**Background and aims:**

Most people who use heroin also smoke tobacco, but there is limited investment to reduce the prevalence of smoking in this group. This may be due to a perception that tobacco is less harmful than other substances and is a lower priority problem. Among people who use heroin in England, we aimed to compare tobacco smoking and illegal drugs in terms of the risk of premature death and years‐of‐life lost.

**Design:**

Period life‐tables based on age‐ and cause‐specific mortality rates. We included six causes of death: (1) smoking‐specific diseases: respiratory cancers and chronic obstructive pulmonary disease, (2) other cancers, (3) cardiovascular diseases, (4) drug poisoning, (5) viral hepatitis and (6) all other causes. We attributed fractions of these deaths to tobacco smoking and illegal drugs.

**Setting:**

England, 2000–2018.

**Participants:**

106 789 people who use illegal opioids, drawn from the Clinical Practice Research Datalink (CPRD).

**Measurements:**

(1) Risk of premature death (before age 70), (2) the proportion of premature deaths attributable to smoking and illegal drugs, (3) the potential reduction in premature deaths by eliminating tobacco and/or illegal drugs. We also estimated years‐of‐life lost in these scenarios, which gives greater weight to deaths at younger ages.

**Findings:**

We estimated that 63.2% [95% confidence interval (CI) = 62.2–64.1] of people who use heroin would die before age 70, compared with 16.2% of the general population. Among people who use heroin, we attributed 23.6% (95% CI = 22.8–24.5) of premature deaths to tobacco smoking and 27.6% (95% CI = 26.7–28.6) to illegal drugs. Elimination of tobacco smoking would have reduced the risk of premature death by 11.8 (95% CI = 10.5–13.1) percentage points to 51.5% (95% CI = 50.6–52.4), while elimination of illegal drugs would have reduced the risk of premature death by 9.4 (95% CI = 7.8–11.0) percentage points to 53.9% (95% CI = 52.7–55.1). Drug‐related deaths typically happened at a younger age than smoking‐related deaths. Therefore, the years‐of‐life lost attributable to illegal drugs (mean per person 4.94 years; 95% CI = 4.71–5.17) were approximately three times greater than for tobacco (mean per person 1.68 years; 95% CI = 1.62–1.75).

**Conclusions:**

Tobacco smoking and illegal drugs each appear to account for around one‐quarter of premature deaths among people who use heroin.

## INTRODUCTION

Prevalence of tobacco smoking is extremely high among people who use heroin and other illegal opioids. For example, in England, 93% of primary care patients who are known to use illegal opioids also currently or previously smoked tobacco [1], and an international systematic review of smoking prevalence among people in treatment for opioid dependence found that an average of 85% currently smoke tobacco [2]. In addition, people who use heroin may have more intensive smoking histories than people in the general population who smoke tobacco [3, 4]. This is reflected in high rates of chronic obstructive pulmonary disease (COPD) and other smoking‐related diseases among people who use heroin [4].

Health promotion and harm reduction activities in this population are focused on reducing the harms of illegal drugs, with tobacco often considered a secondary problem [5]. Although some people who use heroin and other drugs express a desire to quit smoking, there is limited investment and a lack of clear treatment pathways to support quitting in this group. In the United Kingdom (UK) in 2022, only 3% of people who entered treatment programmes for opioid dependence and also smoked tobacco were offered referrals for smoking cessation interventions [6], and in the United States (US) one in five outpatient treatment services for substance use can offer pharmacotherapy to support quitting smoking [7]. One possible reason is that commissioners and staff of these services believe that tobacco is a lower priority than other substances [8]. In the United Kingdom, this may be compounded by financial cuts to substance use services since 2013 [9], which means staff have larger caseloads and spend less time with clients.

We aimed to compare the lifetime risks of smoking and illegal drug use on premature death among people who use illegal opioids. In other words, we aimed to answer the question: ‘if you use heroin and smoke tobacco, which is more likely to kill you?’

## METHODS

### Study design

We used a period life‐table approach [10] to estimate the number of deaths attributable to tobacco smoking and illegal drugs in a cohort of people who use heroin. Period life‐tables assume that mortality rates within age groups observed during a specific time period are experienced across life. This method is often used to estimate period life expectancy, which is the typical age at death under this assumption.

### Source data

This analysis was based on mortality rates in a cohort of people who used illegal opioids drawn from the Clinical Practice Research Datalink [11, 12], which includes pseudonymised patient‐level data from general practice clinical systems in England. The cohort included patients with prescriptions of opioid agonist therapy (methadone or buprenorphine) or clinical observations such as ‘heroin dependence’. Between 1 January 2001 and 30 October 2018, we included 106 789 participants with a median of 8.7 years [interquartile (IQR) = 4.3–13.5] of observation. The median age at cohort entry was 35.1 years (IQR = 29.0–42.3) and 69.1% were male. More detailed demographic characteristics are provided in Data S1. We also accessed mortality records from the Office for National Statistics, from which we extracted the International Statistical Classification of Diseases (ICD‐10) code for the underlying cause of death. We have previously shown that the all‐cause mortality rate in this cohort was 7.72 (95% CI = 7.47–7.97) times higher than in the general population [13]. Heroin was the most commonly used illegal opioid used in England during this period, with 94% of people who injected drugs in 2018 reporting heroin use [14] and few other opioids in the UK illicit drug market. Therefore, this cohort primarily captured people who used heroin.

### Statistical analysis

First, we estimated age‐specific mortality rates for six underlying causes of death: (1) smoking‐specific deaths (respiratory cancers and COPD); (2) other cancers; (3) cardiovascular diseases; (4) drug poisoning; (5) viral hepatitis; and (6) all other causes. ICD‐10 codes for these groups are provided in Data S1. We chose these categories because we considered them to capture the large majority of deaths directly attributable to tobacco smoking or illegal drugs.

Second, we calculated the number of deaths and observation time at each single‐year‐of‐age. For all‐cause deaths and for the six causes of death, we used a Poisson model in which the dependent variable was the count of deaths, and the independent variables were a cubic spline of age with three degrees of freedom and an offset for the logged duration of observation. We used these models to predict mortality rates by age and checked that the sum of modelled cause‐specific mortality rates approximated the modelled all‐cause mortality rate (at all ages the difference was smaller than 2%).

Third, we used life‐tables to estimate the number of all‐cause deaths at each age in a cohort of 100 000, starting at age 18 and ending at age 70. This approach simulated a cohort of people who used heroin and tobacco to the extent these behaviours occurred in our cohort. At each age, we used the ratio of modelled cause‐specific mortality rates to estimate the number of cause‐specific deaths. For comparison, we used the same method to estimate all‐cause deaths in the general population, using an age‐ and sex‐matched sample drawn from the same underlying cohort [15].

Fourth, we assumed that fractions of the six underlying causes of death were attributable to illegal drugs or tobacco smoking. These assumptions were based on epidemiological evidence, with our reasoning provided in Data S1. We assumed that 100% of deaths due to drug poisoning or viral hepatitis in this cohort were attributable to illegal drugs; 100% of deaths due to respiratory cancers or COPD were attributable to tobacco smoking; 50% of deaths due to cardiovascular diseases were attributable to tobacco smoking; and 30% of deaths due to cancers other than respiratory cancers were attributable to tobacco smoking.

Fifth, we reported: (1) the risk of premature death attributable to illegal drugs and tobacco smoking; (2) the reduction in risk of premature death if tobacco smoking and/or illegal drug use was eliminated in this population; and (3) the number of years‐of‐life lost (YLL) in these scenarios, which weighted premature deaths according to the age at death, such that a death at age 69 contributed 0.5 YLLs, at age 68 contributed 1.5 YLLs, etc.

Finally, we estimated CI using a Monte‐Carlo method in which we simulated 1000 datasets with counts of deaths at each age drawn from Poisson distributions, repeated steps 2 to 5 for each simulation and reported the 0.025 and 0.975 quantiles of each parameter to estimate a 95% CI.

As a supplementary analysis, we added assumptions about the effect of stimulants on cardiovascular mortality. Methods and results for this analysis are included in Data S1.

We did analysis using R version 4.4.1 [16], with data and code provided in Data S1. This analysis did not have a pre‐registered analysis plan and should be considered exploratory.

## RESULTS

**FIGURE 1:**
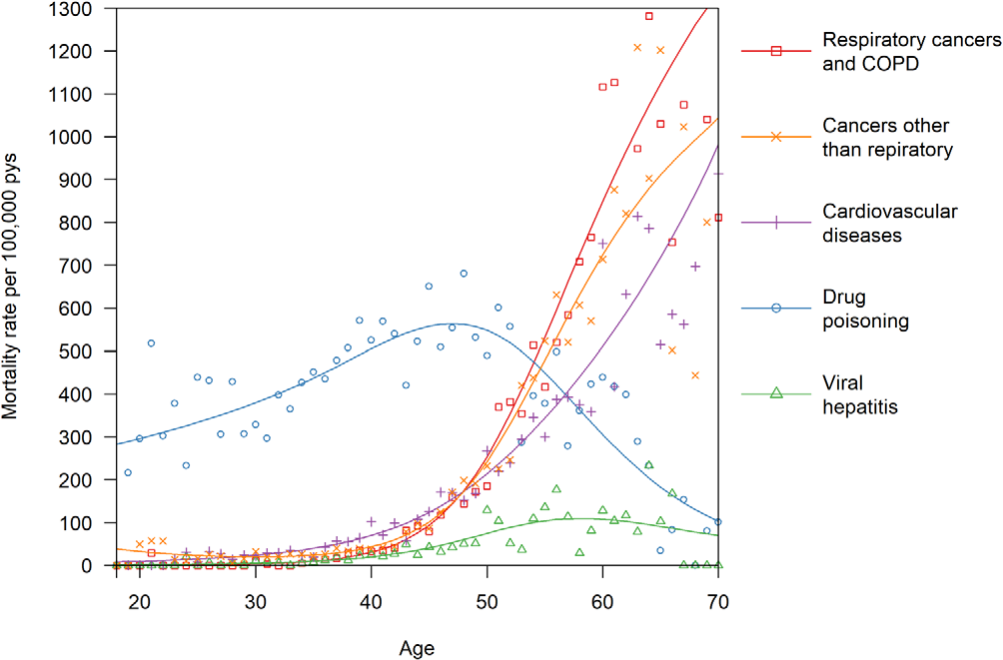
Age‐specific mortality rates among people who used illegal opioids in England, 2001–2018, by major cause of death, observed within age groups (points) and modelled using Poisson regression (lines).

The cohort included 13 010 deaths before age 70. A total of 4373 (33.6%) were due to drug poisoning; 1179 (9.1%) were due to respiratory cancers or COPD; 1168 (9.0%) were due to cancers other than respiratory cancers; 1101 (8.5%) were due to cardiovascular diseases; 303 (2.3%) were due to viral hepatitis; and 4886 (37.6%) were due to other causes. The rate of death due to drug poisoning increased until age 47 and then decreased; the rate of death due to viral hepatitis increased until age 58 and then plateaued; and the rate of death due to respiratory cancers and COPD, other cancers and cardiovascular diseases increased monotonically with age (Figure 1).

**FIGURE 2:**
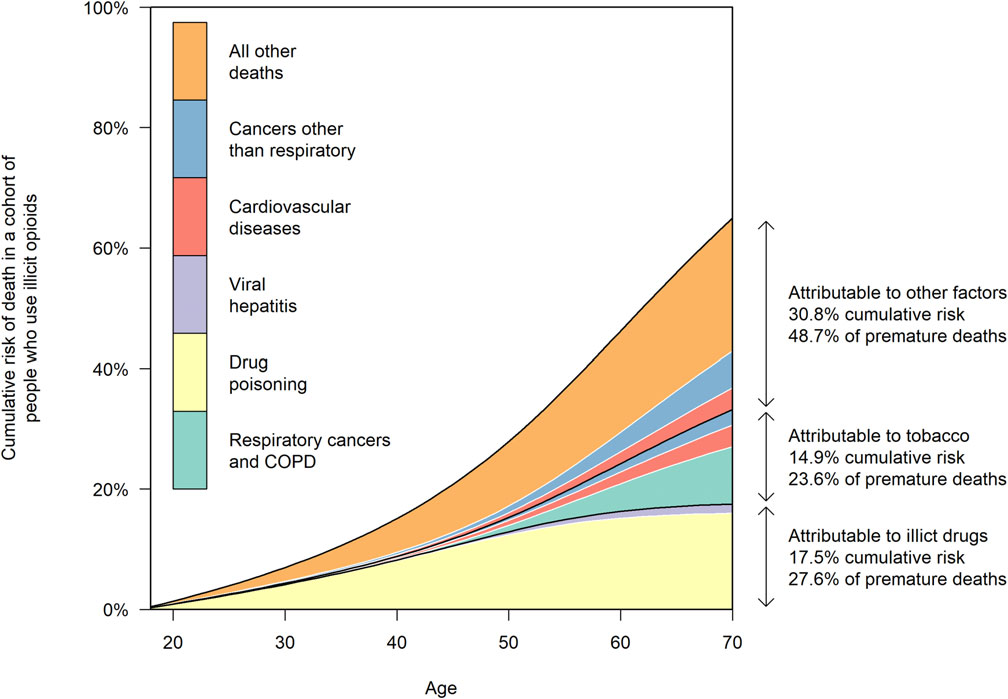
Cumulative risk of death among people who used illegal opioids in England between 2001 and 2018, based on life‐table modelling.

In life‐table modelling, we estimated a 63.2% (95% CI = 62.2–64.1) risk of death before age 70. This compared to 16.2% in the general population. Among people who used illegal opioids, we estimated 17.5% (95% CI = 16.9–18.1) risk of premature death attributable to illegal drugs (27.6% of premature deaths, 95% CI = 26.7–28.6) and 14.9% (95% CI = 14.3–15.5) risk of premature death attributable to tobacco smoking (23.6% of premature deaths, 95% CI = 22.8–24.5) (Figure 2).

Weighting for age at death, we estimated a mean of 12.2 (95% CI = 12.0–12.5) YLLs per person. This compared to 1.9 YLLs in the general population. Among people who used illegal opioids, we estimated 4.9 (95% CI = 4.7–5.2) YLLs attributable to illegal drugs (40.4% of YLLs, 95% CI = 39.0–41.8) and 1.7 (95% CI = 1.6–1.8) YLLs attributable to tobacco smoking (13.8% of YLLs, 95% CI = 13.2–14.4).

In a scenario where illegal drugs were eliminated, we estimated 53.9% (95% CI = 52.7–55.1) risk of death before age 70, representing a risk reduction of 9.4 (95% CI = 7.8–11.0) percentage points (ppts). Note that in this scenario, we estimated 17.5 ppts risk reduction due to prevention of drug poisoning and viral hepatitis, offset by additional deaths due to tobacco smoking and other causes among people who would otherwise have died at a younger age due to drug poisoning or viral hepatitis (Table 1). This is often known as ‘competing risks’.

**TABLE 1:** Risk of premature death among people who used illegal opioids in England between 2001 and 2018, based on observed mortality rates and scenarios where illegal drugs and/or tobacco smoking were eliminated (95% CIs).

	Observed mortality rates	Eliminating illegal drugs	Eliminating tobacco smoking	Eliminating both illegal drugs and tobacco smoking
Risk of death before age 70 (%)				
All	63.2 (62.2–64.1)	53.9 (52.7–55.1)	51.5 (50.6–52.4)	39.1 (38.1–40.1)
Attributable to tobacco	14.9 (14.3–15.5)	18.0 (17.2–18.8)	–	–
Attributable to illegal drugs	17.5 (16.9–18.1)	–	18.0 (17.4–18.7)	–
Others	30.8 (30.0–31.5)	35.9 (35.0–36.7)	33.4 (32.6–34.3)	39.1 (38.1–40.1)
Percent of premature deaths (%)				
All	100	100	100	100
Attributable to tobacco	23.6 (22.8–24.5)	33.4 (32.4–34.6)	–	–
Attributable to illegal drugs	27.6 (26.7–28.6)	–	35.0 (34.0–36.1)	–
Others	48.7 (47.8–49.7)	66.6 (65.4–67.6)	65.0 (63.9–66.0)	100
Change in risk of death before age 70 (ppts)				
All	–	−9.4 (−11.0 to −7.8)	−11.8 (−13.1 to −10.5)	−24.1 (−25.5 to −22.6)
Attributable to tobacco	–	3.1 (2.1–4.1)	−14.9 (−15.5 to −14.3)	−14.9 (−15.5 to −14.3)
Attributable to illegal drugs	–	−17.5 (−18.1 to −16.9)	0.5 (−0.3 to 1.4)	−17.5 (−18.1 to −16.9)
Others	–	5.1 (3.9–6.2)	2.7 (1.6–3.8)	8.3 (7.0–9.6)

Abbreviation: ppts, percentage points.

**FIGURE 3:**
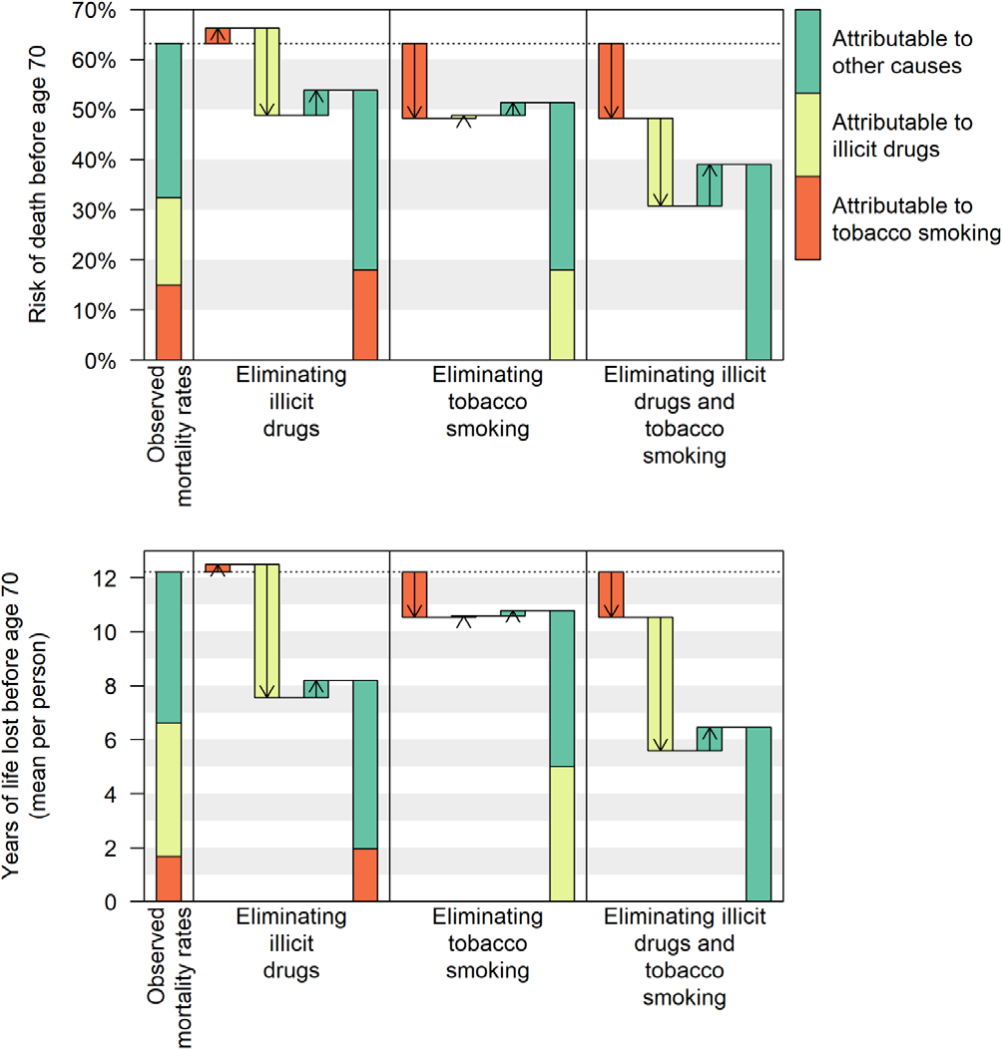
Risk of death and years‐of‐life lost before age 70 among people who used illegal opioids between 2001 and 2018, with risk disaggregated into components attributable to illegal drugs and tobacco smoking, and scenarios where illegal drugs and/or tobacco smoking were eliminated.

In a scenario where tobacco was eliminated, we estimated 51.5% (95% CI = 50.6–52.4) risk of deaths before age 70, representing a risk reduction of 11.8 ppts (95% CI = 10.5–13.1). This comprises 14.9 ppts risk reduction due to prevention of smoking‐attributable diseases, offset by additional deaths due to illegal drugs and other causes (Figure 3).

Although we attributed slightly more premature deaths to illegal drugs, the life table suggested that more premature deaths would be prevented by eliminating tobacco smoking. This is because the rate of smoking‐related deaths increased with age, so that people who did not die from drug poisoning or viral hepatitis remained at increasing risk of smoking‐related death. In contrast, when smoking‐related deaths were prevented, the risk of drug‐related deaths did not increase with age and may have even decreased.

As drug‐related deaths occurred at an earlier age, both the years‐of‐life lost attributable to drugs and the years‐of‐life gained by eliminating drugs were approximately three times greater than those related to tobacco smoking (Table 2).

**TABLE 2:** Years‐of‐life‐lost among people who used illegal opioids in England between 2001 and 2018, based on observed mortality rates and scenarios where illegal drugs and tobacco smoking were eliminated (95% CIs).

	Observed mortality rates	Eliminating illegal drugs	Eliminating tobacco smoking	Eliminating both illegal drugs and tobacco smoking
Years‐of‐life‐lost before age 70 (mean per person)				
All	12.21 (11.97–12.46)	8.19 (8.01–8.39)	10.78 (10.56–11.05)	6.45 (6.28–6.63)
Attributable to tobacco	1.68 (1.62–1.75)	1.96 (1.89–2.04)	–	–
Attributable to illegal drugs	4.94 (4.71–5.17)	–	5.00 (4.80–5.23)	–
Others	5.59 (5.43–5.76)	6.23 (6.06–6.40)	5.78 (5.62–5.95)	6.45 (6.28–6.63)
Percent of years‐of‐life‐lost (%)				
All	100	100	100	100
Attributable to tobacco	13.78 (13.18–14.35)	23.98 (23.16–24.81)	–	–
Attributable to illegal drugs	40.44 (39.01–41.77)	–	46.41 (44.93–47.83)	–
Others	45.77 (44.63–46.98)	76.02 (75.19–76.84)	53.59 (52.17–55.07)	100
Change in years‐of‐life‐lost before age 70 (mean per person; negative values represent years‐of‐life gained)				
All	–	−4.02 (−4.32 to −3.74)	−1.43 (−1.76 to −1.11)	−5.76 (−6.08 to −5.47)
Attributable to tobacco	–	0.28 (0.18–0.38)	−1.68 (−1.75 to −1.62)	−1.68 (−1.75 to −1.62)
Attributable to illegal drugs	–	−4.94 (−5.17 to −4.71)	0.06 (−0.24 to 0.37)	−4.94 (−5.17 to −4.71)
Others	–	0.64 (0.40–0.87)	0.19 (−0.06–0.43)	0.86 (0.61–1.11)

## DISCUSSION

Mortality rates are extremely high among people who use illegal opioids, with two‐thirds dying before age 70. In this study, tobacco smoking and illegal drugs each accounted for approximately 25% of these premature deaths. A similar finding was reported from a cohort study of people in treatment for alcohol dependence in the United States in the 1970s and 1980s, in which 85% either currently or previously smoked tobacco, and more deaths were tobacco‐related than alcohol‐related [17].

Similar to people in the general population who smoke tobacco, a large proportion people who use illegal drugs and also smoke tobacco want to quit smoking [8, 18]. However, there is little investment in smoking cessation for this subgroup [6, 7]. One possible reason is the attitudes and beliefs of staff and commissioners of substance use treatment services. Qualitative research and surveys have found perceptions that people who use illegal drugs do not want to quit, and tobacco has benefits including calming people down and reducing the risk of relapse after clients stop using illegal drugs [19]. Staff often smoke tobacco and may use smoking breaks as an opportunity to build rapport with clients [8, 19].

There is now substantial evidence that these perceptions are false. A systematic review in 2016 identified 35 trials of smoking cessation interventions during substance use treatment and found that nicotine replacement therapy and combined counselling and pharmacotherapy are effective in these settings [20]. The review also found that smoking cessation interventions do not affect abstinence from other drugs, and some evidence suggests that smoking cessation may help people reduce their use of other drugs [21]. Furthermore, smoking cessation is associated with reduced depression, anxiety and improvements in self‐esteem, including among people with a severe mental illness [22].

In addition to smoking cessation interventions using traditional pharmacotherapy [23], e‐cigarettes may be an acceptable and effective tool to support quit attempts among people who use illegal opioids [24]. Interventions including e‐cigarettes are currently being trialled in homeless centres in the United Kingdom [25], and may be effective in services that support people who use illegal drugs.

The strengths of our analysis include: (1) long follow‐up in the underlying cohort study (median 8.7 years), limiting bias in shorter studies that may overestimate the risks of illegal drugs by focusing on participants during periods of heavier drug use; (2) use of national mortality records linked to primary care data, which likely capture almost all deaths; and (3) accounting for competing risks of tobacco smoking and drug‐related deaths in the life‐table method.

Limitations include: (1) lack of generalisability. The mortality rates in our analysis reflected the drug use, intensity of tobacco smoking, and other health risk factors among people who used illegal opioids from 2000 to 2018. Many other contemporary factors also affected these mortality rates, for example, direct acting antivirals for hepatitis C were scaled up the final 2 years of the cohort study underlying this analysis (2017–2018) [26], which may contribute to the reduction in deaths due to viral hepatitis with age, since observation at older ages will be concentrated in later calendar years. The comparative risks of smoking and illegal opioid use are likely to be different in other settings, for example, in North America the risk of death due to drug poisoning may be higher because of synthetic opioids in the drug market [27]. (2) We did not attempt to estimate the impact of cessation of illegal drug use or tobacco smoking. Instead, we estimated the impact of eliminating these risk factors (i.e. assuming people never smoked and/or used drugs), to address the question ‘which is most likely to cause premature death?’ It would be possible to use a similar method to estimate the relative benefits of cessation or interventions to support cessation, by adding parameters regarding the taper in risk after cessation or the effectiveness of interventions. (3) The assumed attributable fractions (e.g. that half of deaths due to cardiovascular disease can be attributed to tobacco smoking) are approximate and the results would be sensitive to these assumptions. (4) We used broad categories of cause of death aiming to capture a large proportion of deaths attributable to illegal drugs and/or tobacco, and these broad categories may have excluded smoking‐ and drug‐attributable deaths. For example, 2.7% of deaths in the underlying cohort were because of pneumonia and influenza [13], some of which could be attributed to tobacco smoking. Similarly, some of the deaths due to respiratory disease may be attributable to smoking of heroin and other drugs. (5) The analysis does not attempt to account for other risk factors such as alcohol use that may contribute to relevant deaths such as those caused by cardiovascular disease, drug poisoning and viral hepatitis. An analysis that estimates the benefits of eliminating multiple risk factors concurrently would likely show smaller effects of each risk factor; and (6) while the analysis accounts for competing risks, there are limitations to the counterfactual assumptions behind this method. For example, if people who died from smoking‐related diseases had survived, we assumed their continued risk of drug‐related death was represented by older people surviving in the cohort. It is possible that individuals with highest risk of drug‐related death tend to die younger, leaving lower‐risk individuals. Therefore, in the scenario where smoking was eliminated, the increase in deaths attributable to drugs may be understated.

## CONCLUSION

Among people who use heroin, tobacco smoking is likely to cause a similar number of premature deaths as illegal drugs.

## AUTHOR CONTRIBUTIONS

**Dan Lewer**: Conceptualization (equal); data curation (equal); formal analysis (equal); methodology (equal); visualisation (equal); writing—original draft presentation (equal); writing—review and editing (equal). **Harry Tattan‐Birch**: Conceptualisation (equal); methodology (equal); writing—original draft presentation (equal); writing—review and editin (equal) **Sharon Cox**: Conceptualisation (equal); methodology (equal); writing—original draft presentation (equal); writing—review and editing (equal).

## DECLARATION OF INTERESTS

D.L. has no competing interests. H.T.B. is a Deputy Statistics and Methodology Editor at the journal *Addiction*. S.C. is a Senior Editor at the journal *Addiction*.

## ETHICS STATEMENT

The study was approved by the Medicines and Healthcare products Regulatory Agency (UK) Independent Scientific Advisory Committee [number 19_142R, under Section 251; National Health Service (NHS) Social Care Act 2006]. This study is based in part on data from the Clinical Practice Research Datalink, obtained under licence from the UK Medicines and Healthcare products Regulatory Agency. The data were provided by patients and collected by the UK NHS as part of their care and support. Individual patient consent is not required for this analysis.

## Supporting information


**Data S1.** Supporting Information.

## Data Availability

The full dataset for this research is provided in Data S1 and at: https://github.com/danlewer/hupio/blob/main/mortality/single-year-of-age-rates/hupio_rates_drugs_vs_smoking_25aug2023.csv. Analysis code is available in Data S1 and at: https://github.com/danlewer/hupio/tree/main/tobacco-life-table.
