# Supplementary material for: Among people who use heroin, tobacco smoking and illegal drugs cause a similar number of premature deaths
Source: Addiction. 2025 Jul 17;120(12):2573–9. doi: 10.1111/add.70140 (PMC12586767; doi:10.1111/add.70140)
Supplement: Supplementary file 1 — Data S1. Supporting Information. [file ADD-120-2573-s001.pdf]

Supplementary material

1. ICD-10 codes for causes of death 2

2. Demographic information about the underlying cohort 4

3. Modelled mortality rates with plots showing 95% confidence intervals 5

4. Smoking attributable fractions 6

5. Dataset (counts of deaths and years of follow-up by opioid status, age, and gender) 9

6. Supplementary analysis including the effect of stimulants on cardiovascular mortality 14

7. Analysis code 16

8. References 24

1. ICD-10 codes for causes of death

Respiratory cancers and chronic obstructive pulmonary disease

- C30-C39 (Malignant neoplasms of respiratory and intrathoracic organs)
- J40 (Bronchitis, not specified as acute or chronic)
- J41 (Simple and mucopurulent chronic bronchitis)
- J42 (Unspecified chronic bronchitis)
- J43 (Emphysema)
- J44 (Other chronic obstructive pulmonary disease)

Other cancers

- C00-C97, except C30-39 (Malignant neoplasms)
- D00-D09 (In situ neoplasms)
- D10-D36 (Benign neoplasms)
- D37-D48 (Neoplasms of uncertain or unknown behaviour

Cardiovascular diseases

- I00-I99 (Diseases of the circulatory system)

Drug poisoning

- F10:F19, except F10 and F17 (Mental and behavioural disorders due to psychoactive substance use)
- X40 (Accidental poisoning by and exposure to nonopioid analgesics, antipyretics and antirheumatics)
- X41 (Accidental poisoning by and exposure to antiepileptic, sedative-hypnotic, antiparkinsonism and psychotropic drugs, not elsewhere classified)
- X42 (Accidental poisoning by and exposure to narcotics and psychodysleptics [hallucinogens], not elsewhere classified)
- X43 (Accidental poisoning by and exposure to other drugs acting on the autonomic nervous system)
- X44 (Accidental poisoning by and exposure to other and unspecified drugs, medicaments and biological substances)
- X60 (Intentional self-poisoning by and exposure to nonopioid analgesics, antipyretics and antirheumatics)
- X61 (Intentional self-poisoning by and exposure to antiepileptic, sedative-hypnotic, antiparkinsonism and psychotropic drugs, not elsewhere classified)
- X62 (Intentional self-poisoning by and exposure to narcotics and psychodysleptics [hallucinogens], not elsewhere classified)
- X63 (Intentional self-poisoning by and exposure to other drugs acting on the autonomic nervous system)
- X64 (Intentional self-poisoning by and exposure to other and unspecified drugs, medicaments and biological substances)
- X85 (Assault by drugs, medicaments and biological substances)

- Y10 (Poisoning by and exposure to nonopioid analgesics, antipyretics and antirheumatics, undetermined intent)
- Y11 (Poisoning by and exposure to antiepileptic, sedative-hypnotic, antiparkinsonism and psychotropic drugs, not elsewhere classified, undetermined intent)
- Y12 (Poisoning by and exposure to narcotics and psychodysleptics [hallucinogens], not elsewhere classified, undetermined intent)
- Y13 (Poisoning by and exposure to other drugs acting on the autonomic nervous system, undetermined intent)
- Y14 (Poisoning by and exposure to other and unspecified drugs, medicaments and biological substances, undetermined intent)

Viral hepatitis

- B15:19 (Viral hepatitis)

2. Demographic information about the underlying cohort

**Variable**	**Level**	**Number (%)**
Total		106,789 (100.0)
Date of cohort entry	2001-2003	25,229 (23.6)
	2004-2006	15,429 (14.4)
	2007-2009	17,716 (16.6)
	2010-2012	17,635 (16.5)
	2013-2015	16,688 (15.6)
	2016-2018	14,092 (13.2)
Years of follow-up	Median [IQR]	8.7 [4.3-13.5]
Age at entry	Median [IQR]	35.1 [29.0-42.3]
Sex	Male	73,791 (69.1)
	Female	32,998 (30.9)
Region of residence at entry	North West	22,274 (20.9)
	South West	20,187 (18.9)
	West Midlands	16,910 (15.8)
	London	14,694 (13.8)
	South Central	9,699 (9.1)
	South East Coast	5,668 (5.3)
	North East	5,581 (5.2)
	Yorkshire & The Humber	4,844 (4.5)
	East of England	4,640 (4.3)
	East Midlands	2,243 (2.1)
	Missing	49 (<0.1)
Index of Multiple Deprivation of home address at study entry	1: Least deprived	7,412 (6.9)
	2	11,361 (10.6)
	3	16,339 (15.3)
	4	26,090 (24.4)
	5: Most deprived	45,396 (42.5)
	Missing	191 (0.2)
Ethnicity*	White (British/Irish/Other)	93,445 (87.5)
	Mixed/Multiple ethnic groups	1,416 (1.3)
	Asian/Asian British	2,489 (2.3)
	Black/African/Caribbean/Black British	2,378 (2.2)
	Other ethnic group	1,601 (1.5)
	Unknown	5,460 (5.1)
Smoking status at entry†	Never	7,295 (6.8)
	Ex	7,011 (6.6)
	Current	83,483 (78.2)
	Missing	9,000 (8.4)
Body Mass Index at entry (kg/m^2)	Underweight (<18.5)	5,468 (5.1)
	Healthy (18.5-24.9)	44,480 (41.7)
	Overweight (25-29.9)	20,300 (19.0)
	Obese (30-39.9)	11,087 (10.4)
	Severely obese (40+)	1,762 (1.6)
	Missing	23,692 (22.2)
Died during follow-up		13,209 (12.4)

IQR = Interquartile range

* Ethnicity is derived from primary care, hospital admission, and hospital outpatient records. The mostly commonly recorded value is used, or where values are tied the most recently recorded tied value is used. The opioid group has less missing data due to higher rates of hospital admission.

† Smoking status is derived from the most recent relevant record in primary care data prior to cohort entry.

3. Modelled mortality rates with plots showing 95% confidence intervals

Figure S1: all-cause and cause-specific mortality rates among people who use illegal opioids in England, 2000-2018, observed (points) and modelled using Poisson regression (lines), with 95% confidence intervals in shaded bands

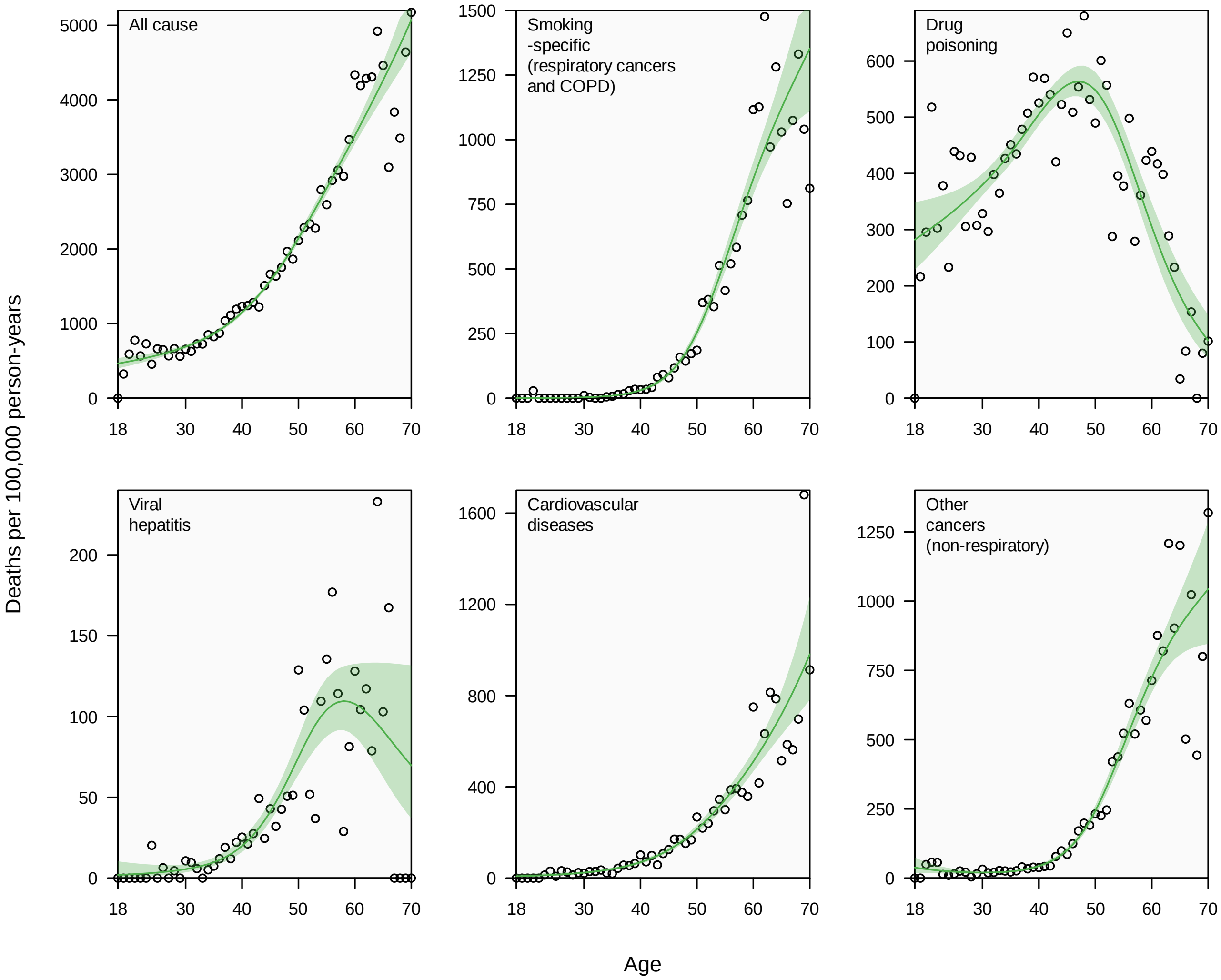

4. Smoking attributable fractions

The population attributable fraction (ie. the proportion of cases that can be attributed to a risk factor) can be calculated from the prevalence of a risk factor and the relative risk of the risk factor on the outcome. The formula is:[1]

$$\text{Population Attributable Fraction} = \frac{\text{Prevalence x (Relative Risk-1)}}{\text{Prevalence x (Relative Risk-1)+1}}$$

Among invididuals exposed to the risk factor, this equation simplifes to:

$$\text{Attributable fraction in exposed} = 1 - \frac{1}{\text{Relative Risk}}$$

For example, if 20% of the population smoke tobacco, and smoking causes the risk of lung cancer to increase 20-fold, then 80% of lung cancer cases in the population could be attributed to tobacco smoking. Among smokers, 95% of lung cancer cases could be attributed to tobacco smoking.

In our cohort, all participants use illegal opioids, and almost all smoke tobacco. We therefore aimed to identify estimates of the relative risks of illegal opioids and tobacco smoking on cause-specific death, rather than estimates of the prevalence of these risk factors among people who use illegal opioids.

We identified the following estimates:

Drug poisoning and viral hepatitis

In a cohort study in England (the same cohort as that underlying the present study), a comparison between participants with a history of using illegal opioids and age/sex-matched participants from the general population:[2]

- The relative risk of death due to drug poisoning was 52.07 (95% CI 46.43-59.48), implying an attributable risk of 98%
- The relative risk of death due to viral hepatitis was 103.50 (95% CI 61.74-242.64), implying an attributable risk of 99%

For deaths due to drug poisoning and viral hepatitis, we assumed that 100% of deaths were attributable to illegal drug use.

Respiratory cancers

A report on the British Doctors' Study includes age-standardised mortality rates (ASMR, per 1000 years) for selected causes of death and by smoking status.[3]

- Cancers of the lung: ASMR for never-smokers = 0.17; ASMR for current smokers (25+/day) = 4.17; Relative risk = 24.5, implying attributable risk of 96%
- Cancers of the mouth, pharynx, larynx, oesophagus: ASMR for never-smokers = 0.09; ASMR for current smokers (25+/day) = 1.06; Relative risk = 11.8, implying attributable risk of 92%

A meta-analysis in 2016 included 19 cohort studies estimating the effect of current smoking on site-specific cancers:[4]

- The pooled hazard ratio for current smoking on lung cancer was 13.1, implying attributable risk of 92%

We assumed that 100% of deaths due to respiratory cancers were attributable to tobacco smoking.

Chronic obstructive pulmonary disease

In the British Doctors' Study:[3]

- ASMR for never-smokers = 0.11; ASMR for current smokers (25+/day) = 2.61; Relative risk = 23.7, implying attributable risk of 96%

We assumed that 100% of deaths due to COPD were attributable to tobacco smoking.

Other cancers

In the British Doctors' Study:[3]

- All cancers except cancers of the lung, mouth, pharynx, larynx, oesophagus: ASMR for never-smokers = 3.34; ASMR for current smokers (25+/day) = 5.38; Relative risk = 1.6, implying attributable risk of 38%

In the 2016 meta-analysis, hazard ratios for current smoking on cancers other than lung cancer were:[4]

- Colorectal: 1.20
- Gastric: 1.74
- Pancreatic: 1.90
- Breast: 1.07
- Prostate: 0.81
- The mean of logged hazard ratios for these cancers is 0.25, which gives a mean hazard ratio of 1.28, implying an attributable risk of 22%

We assumed that 30% of deaths due to cancers other than respiratory cancers were attributable to tobacco smoking.

Cardiovascular diseases

In the British Doctors' Study:[3]

- Ischaemic heart disease: ASMR for never-smokers = 6.19; ASMR for current smokers (25+/day) = 11.11; Relative risk = 1.79, implying attributable risk of 44%
- Cerebrovascular disease: ASMR for never-smokers = 2.75; ASMR for current smokers (25+/day) = 5.23; Relative risk = 1.90, implying attributable risk of 47%
- Other vascular (including respiratory heart) disease: ASMR for never-smokers = 2.83; ASMR for current smokers (25+/day) = 5.33; Relative risk = 1.88, implying attributable risk of 47%

- Total across these three groups: ASMR for never-smokers = 11.77; ASMR for current smokers (25+/day) = 21.67; Relative risk = 1.84, implying attributable risk of 46%

We assumed that 50% of deaths due to cardiovascular diseases were attributable to tobacco smoking.

Other diseases

We assumed that no deaths due to diseases with an underlying cause not listed above were attributable to either tobacco or illegal drugs (ie. the attributable fractions were 0%).

5. Dataset (counts of deaths and years of follow-up by opioid status, age, and gender)

exposure	ag	gender	svd_smk	svd_cvd	svd_otc	svd_drd	svd_hep	svd_oth	fu	total
FALSE	18	female	0	0	0	0	0	1	1045.1	1
TRUE	18	female	0	0	0	0	0	0	93.711	0
FALSE	18	male	0	0	0	0	0	1	1381.6	1
TRUE	18	male	0	0	0	0	0	0	123.92	0
FALSE	19	female	0	0	0	0	0	0	1843.6	0
TRUE	19	female	0	0	0	0	0	0	416.16	0
FALSE	19	male	0	0	1	0	0	0	2676.9	1
TRUE	19	male	0	0	0	2	0	1	508.61	3
FALSE	20	female	0	0	1	0	0	0	3065.6	1
TRUE	20	female	0	0	1	2	0	1	875.04	4
FALSE	20	male	0	0	0	0	0	6	4569.7	6
TRUE	20	male	0	0	0	4	0	4	1155	8
FALSE	21	female	0	0	0	0	0	0	4752.9	0
TRUE	21	female	1	0	0	2	0	3	1449.3	6
FALSE	21	male	0	0	0	1	0	2	7216.6	3
TRUE	21	male	0	0	2	16	0	3	2026.3	21
FALSE	22	female	0	0	0	0	0	2	6753.9	2
TRUE	22	female	0	0	2	2	0	4	2167.6	8
FALSE	22	male	0	0	0	1	0	5	10461	6
TRUE	22	male	0	0	1	14	0	7	3122.7	22
FALSE	23	female	0	0	0	0	0	1	9028.3	1
TRUE	23	female	0	0	1	5	0	10	2947.3	16
FALSE	23	male	0	1	0	0	0	5	14311	6
TRUE	23	male	0	1	0	23	0	14	4457.5	38
FALSE	24	female	0	0	1	2	0	3	11602	6
TRUE	24	female	0	1	1	8	0	3	3821.7	13
FALSE	24	male	1	1	0	4	0	10	18918	16
TRUE	24	male	0	2	0	15	2	13	6049.3	32
FALSE	25	female	0	0	0	0	0	1	14313	1
TRUE	25	female	0	0	0	8	0	7	4732.9	15
FALSE	25	male	0	1	1	0	0	15	24114	17
TRUE	25	male	0	1	2	47	0	18	7792.8	68
FALSE	26	female	0	0	2	0	0	1	17187	3
TRUE	26	female	0	1	1	18	0	4	5753.9	24
FALSE	26	male	0	0	0	2	0	27	29953	29
TRUE	26	male	0	4	3	49	1	20	9765.5	77
FALSE	27	female	0	0	4	1	0	4	20074	9
TRUE	27	female	0	1	1	11	0	4	6746	17
FALSE	27	male	0	3	5	0	0	19	36300	27
TRUE	27	male	0	4	3	46	0	36	11906	89
FALSE	28	female	0	0	2	1	0	4	23046	7
TRUE	28	female	0	2	1	22	0	9	7774.5	34
FALSE	28	male	0	2	4	6	0	17	43037	29
TRUE	28	male	0	1	0	72	1	38	14157	112
FALSE	29	female	0	0	2	1	0	5	25808	8
TRUE	29	female	0	3	3	17	0	16	8660.4	39
FALSE	29	male	0	1	5	6	0	20	49781	32
TRUE	29	male	0	3	1	60	0	38	16390	102
FALSE	30	female	0	2	1	1	0	6	28468	10
TRUE	30	female	3	2	3	25	1	17	9429.6	51
FALSE	30	male	0	1	4	9	0	27	56495	41
TRUE	30	male	0	4	6	67	2	54	18579	133
FALSE	31	female	0	2	5	0	0	9	30820	16
TRUE	31	female	0	2	3	21	0	24	10219	50
FALSE	31	male	1	7	3	7	0	35	63274	53

exposure	ag	gender	svd_smk	svd_cvd	svd_otc	svd_drd	svd_hep	svd_oth	fu	total
TRUE	31	male	1	7	3	71	3	60	20803	145
FALSE	32	female	0	1	1	1	0	9	32991	12
TRUE	32	female	0	3	3	31	0	22	10921	59
FALSE	32	male	1	1	7	8	0	34	70035	51
TRUE	32	male	0	7	4	104	2	71	22989	188
FALSE	33	female	0	0	7	2	0	4	34630	13
TRUE	33	female	0	4	7	29	0	36	11467	76
FALSE	33	male	1	4	8	8	0	40	76110	61
TRUE	33	male	0	9	3	104	0	73	24988	189
FALSE	34	female	0	1	5	2	0	7	35942	15
TRUE	34	female	2	2	2	33	1	44	11943	84
FALSE	34	male	3	4	11	11	0	45	81615	74
TRUE	34	male	0	7	8	132	1	97	26730	245
FALSE	35	female	0	8	6	3	0	4	36823	21
TRUE	35	female	1	5	5	43	0	42	12116	96
FALSE	35	male	0	7	10	10	0	51	86146	78
TRUE	35	male	2	3	4	139	3	86	28229	237
FALSE	36	female	1	0	6	3	0	8	37452	18
TRUE	36	female	3	9	5	39	1	48	12192	105
FALSE	36	male	1	17	7	7	0	38	89810	70
TRUE	36	male	3	9	6	142	4	94	29444	258
FALSE	37	female	0	7	8	3	0	12	37255	30
TRUE	37	female	3	11	8	39	1	56	12102	118
FALSE	37	male	4	14	19	15	0	43	91619	95
TRUE	37	male	4	13	9	162	7	123	29910	318
FALSE	38	female	2	6	7	5	0	11	36455	31
TRUE	38	female	4	10	6	43	0	53	11808	116
FALSE	38	male	1	10	11	12	0	52	92076	86
TRUE	38	male	8	13	8	168	5	145	29787	347
FALSE	39	female	1	6	11	2	0	15	35278	35
TRUE	39	female	3	4	4	60	5	51	11334	127
FALSE	39	male	1	21	15	12	0	59	91164	108
TRUE	39	male	11	22	12	172	4	137	29283	358
FALSE	40	female	1	5	7	0	0	10	34021	23
TRUE	40	female	4	15	7	51	4	51	10837	132
FALSE	40	male	4	9	15	16	0	56	89884	100
TRUE	40	male	9	25	8	156	6	149	28557	353
FALSE	41	female	0	7	12	2	0	6	32640	27
TRUE	41	female	5	7	8	43	3	49	10290	115
FALSE	41	male	3	16	17	9	1	55	87630	101
TRUE	41	male	8	20	8	172	5	141	27487	354
FALSE	42	female	2	2	14	1	0	12	31267	31
TRUE	42	female	8	7	4	46	4	41	9758.4	110
FALSE	42	male	7	20	28	9	0	80	85251	144
TRUE	42	male	7	29	12	150	6	152	26509	356
FALSE	43	female	2	3	15	2	0	8	29853	30
TRUE	43	female	8	2	7	31	3	43	9187	94
FALSE	43	male	8	31	20	9	0	65	81904	133
TRUE	43	male	20	18	20	114	14	142	25290	328
FALSE	44	female	1	4	24	3	0	10	28258	42
TRUE	44	female	9	9	10	25	3	50	8639.5	106
FALSE	44	male	6	29	23	5	2	73	78225	138
TRUE	44	male	21	26	22	145	5	166	23884	385
FALSE	45	female	8	3	13	0	0	15	26461	39
TRUE	45	female	11	6	8	37	5	44	8117.2	111
FALSE	45	male	9	24	28	4	0	71	73607	136
TRUE	45	male	13	32	18	160	8	162	22190	393
FALSE	46	female	2	5	8	2	0	14	24909	31
TRUE	46	female	8	14	9	31	2	57	7553.1	121

exposure	ag	gender	svd_smk	svd_cvd	svd_otc	svd_drd	svd_hep	svd_oth	fu	total
FALSE	46	male	2	36	26	11	0	76	68492	151
TRUE	46	male	25	34	26	112	7	135	20548	339
FALSE	47	female	2	3	10	3	0	13	23214	31
TRUE	47	female	13	11	20	34	2	35	6990.3	115
FALSE	47	male	9	34	35	3	0	77	62997	158
TRUE	47	male	28	33	24	109	9	135	18831	338
FALSE	48	female	2	2	13	1	0	10	21518	28
TRUE	48	female	9	12	19	39	2	42	6466.9	123
FALSE	48	male	8	37	28	3	2	65	57674	143
TRUE	48	male	25	24	28	122	10	134	17204	343
FALSE	49	female	2	5	16	1	0	12	19734	36
TRUE	49	female	16	2	15	25	1	30	5937.4	89
FALSE	49	male	17	32	37	3	3	62	52177	154
TRUE	49	male	21	34	26	89	10	131	15516	311
FALSE	50	female	6	7	21	1	0	14	18097	49
TRUE	50	female	14	16	7	17	2	53	5447.4	109
FALSE	50	male	8	31	34	4	1	63	47244	141
TRUE	50	male	22	36	38	78	23	104	13953	301
FALSE	51	female	1	4	12	1	0	13	16573	31
TRUE	51	female	12	12	15	23	3	35	4952.1	100
FALSE	51	male	17	31	36	2	0	50	42563	136
TRUE	51	male	52	26	24	81	15	98	12356	296
FALSE	52	female	2	6	20	0	0	12	15048	40
TRUE	52	female	18	4	12	27	0	27	4484	88
FALSE	52	male	15	31	26	0	0	41	38023	113
TRUE	52	male	41	33	26	59	8	106	10955	273
FALSE	53	female	0	6	16	4	0	8	13576	34
TRUE	53	female	15	10	22	14	0	27	3979.9	88
FALSE	53	male	15	22	28	1	0	54	33386	120
TRUE	53	male	33	30	35	25	5	93	9574.6	221
FALSE	54	female	8	4	15	1	0	16	12142	44
TRUE	54	female	23	10	15	11	0	31	3524.5	90
FALSE	54	male	12	37	31	2	0	49	29269	131
TRUE	54	male	38	31	37	36	13	87	8351.9	242
FALSE	55	female	5	2	17	0	0	9	10847	33
TRUE	55	female	4	9	23	7	4	25	3136.4	72
FALSE	55	male	19	40	34	4	0	35	25641	132
TRUE	55	male	39	22	31	32	10	62	7190.6	196
FALSE	56	female	7	4	16	1	0	9	9747.5	37
TRUE	56	female	9	12	23	7	5	17	2823.8	73
FALSE	56	male	18	32	32	2	0	38	22416	122
TRUE	56	male	38	23	34	38	11	47	6214.8	191
FALSE	57	female	3	3	13	1	0	14	8754.3	34
TRUE	57	female	15	11	13	4	2	24	2512.6	69
FALSE	57	male	15	29	32	0	0	34	19516	110
TRUE	57	male	31	20	28	18	7	68	5367.9	172
FALSE	58	female	2	7	18	0	0	16	7948.9	43
TRUE	58	female	11	5	14	4	0	15	2270.9	49
FALSE	58	male	15	20	37	0	0	28	17116	100
TRUE	58	male	38	21	28	21	2	47	4649.3	157
FALSE	59	female	7	5	15	1	0	12	7239.7	40
TRUE	59	female	14	6	9	11	2	14	2069.8	56
FALSE	59	male	10	18	39	0	0	17	15124	84
TRUE	59	male	33	16	26	15	3	64	4073	157
FALSE	60	female	6	7	17	0	0	13	6601.3	43
TRUE	60	female	21	10	10	8	2	12	1869.9	63
FALSE	60	male	14	24	24	2	0	28	13452	92
TRUE	60	male	40	31	29	16	5	53	3595.2	174
FALSE	61	female	8	6	16	1	0	11	6071.9	42

exposure	ag	gender	svd_smk	svd_cvd	svd_otc	svd_drd	svd_hep	svd_oth	fu	total
TRUE	61	female	23	8	18	3	1	12	1675.6	65
FALSE	61	male	25	26	24	1	0	17	11890	93
TRUE	61	male	31	12	24	17	4	48	3118.8	136
FALSE	62	female	6	5	13	0	0	10	5640.8	34
TRUE	62	female	24	7	10	6	0	4	1530	51
FALSE	62	male	15	31	29	0	0	35	10569	110
TRUE	62	male	39	20	25	11	5	32	2736.9	132
FALSE	63	female	5	4	19	0	0	13	5146.1	41
TRUE	63	female	11	7	9	4	2	10	1421.8	43
FALSE	63	male	21	20	43	0	0	23	9293.6	107
TRUE	63	male	26	24	37	7	1	26	2385.1	121
FALSE	64	female	10	8	12	0	0	5	4645.7	35
TRUE	64	female	17	6	11	0	0	18	1345.2	52
FALSE	64	male	16	18	22	0	0	24	8135.1	80
TRUE	64	male	27	21	20	8	8	33	2088.6	117
FALSE	65	female	5	6	11	0	0	4	4143.8	26
TRUE	65	female	15	5	11	0	1	19	1173.1	51
FALSE	65	male	15	25	27	1	0	25	6971.8	93
TRUE	65	male	15	10	24	1	2	27	1740.1	79
FALSE	66	female	3	2	14	0	0	10	3654.4	29
TRUE	66	female	5	7	2	2	0	11	975.25	27
FALSE	66	male	15	18	22	1	0	17	5909.8	73
TRUE	66	male	13	7	10	0	4	13	1414.5	47
FALSE	67	female	15	4	14	0	0	4	3149.2	37
TRUE	67	female	12	3	7	2	0	8	816.61	32
FALSE	67	male	15	14	22	0	0	20	4920.1	71
TRUE	67	male	9	8	13	1	0	12	1137.8	43
FALSE	68	female	9	8	10	2	0	4	2666.1	33
TRUE	68	female	10	4	1	0	0	2	669.7	17
FALSE	68	male	5	10	19	0	0	16	4006.3	50
TRUE	68	male	11	7	6	0	0	14	907.84	38
FALSE	69	female	1	3	7	0	0	8	2206.9	19
TRUE	69	female	7	8	3	0	0	4	541.55	22
FALSE	69	male	13	10	8	1	0	17	3188.1	49
TRUE	69	male	6	13	7	1	0	9	708.03	36
FALSE	70	female	1	3	7	0	0	9	1825.4	20
TRUE	70	female	5	2	5	1	0	8	436.84	21
FALSE	70	male	7	20	12	0	1	14	2513.8	54
TRUE	70	male	3	7	8	0	0	12	548.65	30
FALSE	71	female	2	5	5	0	0	6	1492.2	18
TRUE	71	female	2	2	1	0	0	4	367.6	9
FALSE	71	male	5	11	13	0	0	9	1876.4	38
TRUE	71	male	7	5	3	1	0	4	420.12	20
FALSE	72	female	2	4	5	0	0	1	1200.1	12
TRUE	72	female	4	1	3	0	0	2	281.86	10
FALSE	72	male	7	2	13	0	0	7	1403.6	29
TRUE	72	male	8	4	0	0	0	5	329.05	17
FALSE	73	female	6	2	6	0	0	6	960.99	20
TRUE	73	female	4	2	0	0	0	3	215.65	9
FALSE	73	male	5	3	3	0	0	6	1082.3	17
TRUE	73	male	1	6	2	0	0	3	252.23	12
FALSE	74	female	2	1	4	0	0	2	748.72	9
TRUE	74	female	3	0	1	0	0	3	159.62	7
FALSE	74	male	2	8	7	0	0	5	831.78	22
TRUE	74	male	2	4	2	0	0	7	177.31	15
FALSE	75	female	0	5	4	0	0	3	560.4	12
TRUE	75	female	1	1	0	0	0	2	126.21	4
FALSE	75	male	6	6	6	0	0	4	620.76	22
TRUE	75	male	2	1	2	0	0	3	129.75	8

exposure	ag	gender	svd_smk	svd_cvd	svd_otc	svd_drd	svd_hep	svd_oth	fu	total
FALSE	76	female	1	2	5	0	0	6	419.57	14
TRUE	76	female	0	0	1	0	0	2	91.702	3
FALSE	76	male	4	3	3	0	0	5	434.23	15
TRUE	76	male	3	2	2	0	0	3	86.522	10
FALSE	77	female	1	1	3	0	0	1	318.18	6
TRUE	77	female	0	0	0	0	0	1	64.98	1
FALSE	77	male	0	4	5	0	0	2	308.51	11
TRUE	77	male	3	2	0	0	0	1	60.419	6
FALSE	78	female	2	2	3	0	0	1	239.21	8
TRUE	78	female	2	0	0	0	0	1	51.797	3
FALSE	78	male	0	1	1	0	0	0	219.83	2
TRUE	78	male	0	0	0	0	0	2	38.79	2
FALSE	79	female	0	3	0	0	0	1	177.82	4
TRUE	79	female	0	0	0	0	0	4	39.658	4
FALSE	79	male	0	1	2	0	0	4	154.6	7
TRUE	79	male	0	1	0	0	0	2	27.592	3
FALSE	80	female	4	2	8	0	0	6	358.74	20
TRUE	80	female	0	2	0	0	0	1	74.675	3
FALSE	80	male	2	4	3	0	0	7	218.24	16
TRUE	80	male	0	0	2	0	0	0	33.202	2

- exposure = whether the participant has a history of using illicit opioids (TRUE = yes; FALSE = no)
- ag = single year of age at last birthday
- gender = gender recorded in primary care records
- svd_smk = count of deaths with an underlying cause of respiratory cancer or COPD
- svd_cvd = count of deaths with an underlying cause of cardiovascular disease
- svd_otc = count of deaths with an underlying cause of cancers other than respiratory cancers
- svd_drd = count of deaths with an underlying cause of drug poisoning
- svd_hep = count of deaths with an underlying cause of viral hepatitis
- svd_oth = count of deaths with an all other underlying causes
- fu = years of observation
- total = total deaths

6. Supplementary analysis including the effect of stimulants on cardiovascular mortality

Our analysis did not account for the cardiovascular risks of illegal stimulants use and may therefore have underestimated the number of premature deaths attributable to illegal drugs.

Many people who use heroin and other illegal opioids also use stimulants.[5,6] In the UK, crack cocaine is the primary stimulant used by people who use heroin, though the prevalence of powder cocaine use is increasing in this population.[7] Crack cocaine is usually either injected or smoked, either alone or in combination with heroin and other drugs.

Based on a capture-recapture estimate of the number of people using opiates and/or crack cocaine in England in 2019-2020, there were 129,584 individuals using both opiates and crack cocaine and 164,279 individuals using opiates only.[8] This suggests that 44% of people who use opiates also use crack cocaine. Based on a cross-sectional survey of people who inject drugs in England, Wales, and Northern Ireland in 2023; 90% injected heroin in the past 4 weeks and 53% injected crack cocaine.[7] The proportion of people who use opiates that also use crack cocaine cannot be calculated directly from these latter values, though given the vast majority of this sample report heroin use the data are likely to suggest that around half also use crack cocaine.

Illegal stimulants have well established cardiovascular risks.[9–12] These risks are both acute (such as myocardial infarction, stroke, and arrythmias after use of drugs) and chronic (such as anatomical changes leading to lower ejection fractions and heart failure). There is limited epidemiological evidence to quantify the effect of stimulant use on cardiovascular mortality among people who use opioids. In a cohort study of people entering treatment for cocaine and opioid use disorder in Barcelona between 1997 and 2001, the rate of death due to cardiovascular diseases among those using cocaine only was 3.5 (95% CI 2.3–5.3) times the rate in the general population.[13] This is likely to be an upper limit on the effect of regular cocaine use on cardiovascular mortality because the raised cardiovascular mortality rate also reflects other risk factors, particularly tobacco smoking, that are not adjusted in the analysis.

If we assume that (a) 50% of people who use illegal opioids are also regular stimulant users; and (b) the risk ratio for stimulants on cardiovascular mortality is 3x; then we can calculate the population attributable fraction as $(0.5 * (3-1)) / (0.5 * (3-1) + 1) = 0.5$. This likely represents an upper limit for the proportion of cardiovascular deaths in the population that can be attributed to illegal drug use. We re-ran the analysis assuming a range of population attributable fractions from 0 to 0.5. The results are shown in the table below, and figure S2 shows the cumulative deaths by age where 0.3 of cardiovascular deaths are attributed to illegal drug use.

Table: cumulative risk of death due to tobacco smoking, illegal drugs, and other risk factors; under different assumptions of the proportion of cardiovascular deaths attributable to illegal drugs

Percentage of cardiovascular deaths attributable to illegal drug use	Cumulative risk of premature death			
	All cause	Attributable to tobacco smoking	Attributable to illegal drugs	Attributable to other factors
0 (main analysis)	63.2	14.9	17.5	30.8
5	63.2	14.9	17.8	30.5
10	63.2	14.9	18.2	30.1
15	63.2	14.9	18.5	29.8
20	63.2	14.9	18.8	29.4
25	63.2	14.9	19.2	29.1
30 (shown in chart below)	63.2	14.9	19.5	28.7
35	63.2	14.9	19.9	28.4
40	63.2	14.9	20.2	28.1
45	63.2	14.9	20.5	27.7
50	63.2	14.9	20.9	27.4

Figure S2: cumulative risk of premature death by age, assuming that 30% of cardiovascular deaths are attributable to illegal drugs

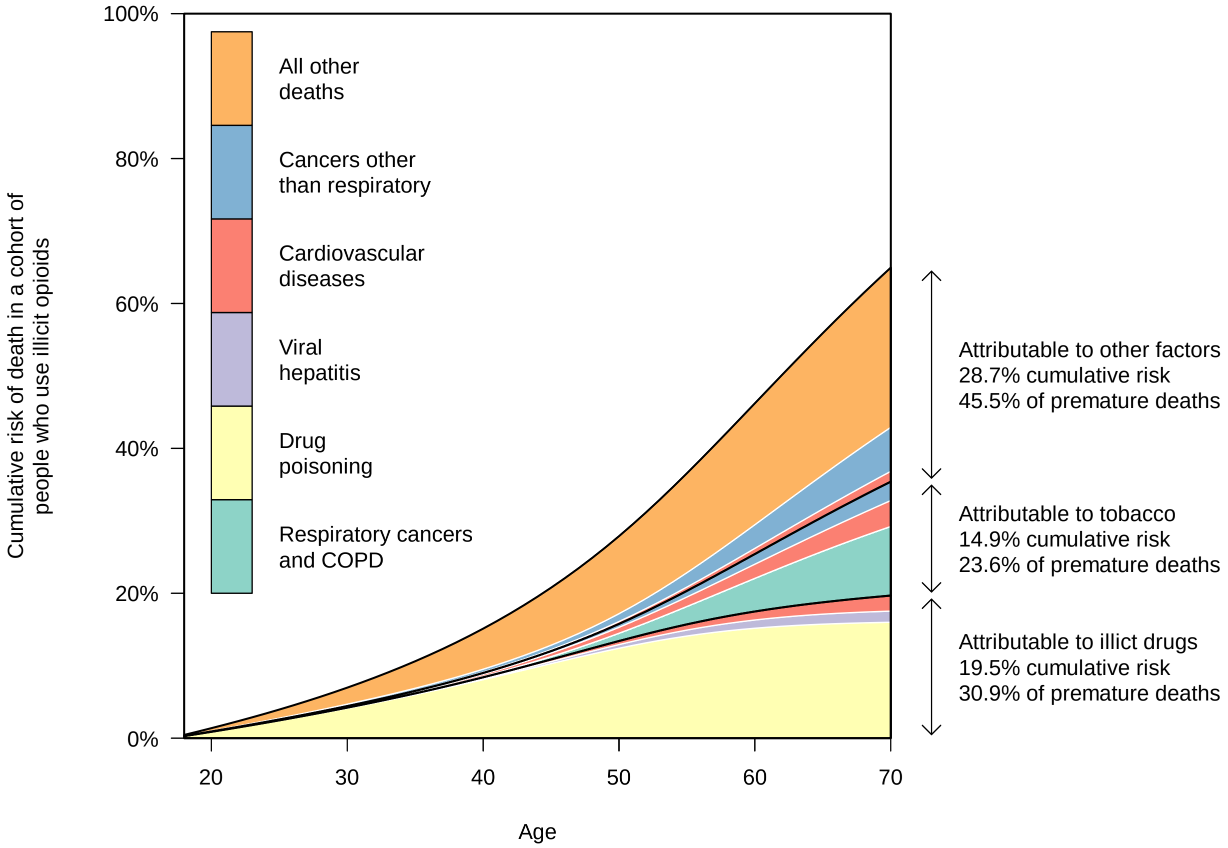

7. Analysis code

Also available at: https://github.com/danlewer/hupio/tree/main/tobacco-life-table

```
options(scipen = 999)

# ===============================
# libraries and general functions
# -------------------------------

library(data.table)
library(splines)
library(RColorBrewer)
library(devEMF) # for enhanced metafile (Windows vector graphic)

# functions to assist plotting

roundup <- function (x, dig = 2) {
  l <- floor(log10(x)) + 1
  r <- ceiling(x / (10 ^ (l-dig)))
  r * 10 ^ (l-dig)
}

yax <- function (x, tickabove = F, ntick = 5) { # make y-axis
  l <- c(c(1, 2, 4, 5, 25) %o% 10^(0:8))
  d <- l[which.min(abs(x/ntick - l))]
  d <- 0:(ntick+1) * d
  i <- findInterval(x, d)
  if (tickabove) {i <- i + 1}
  d[seq_len(i)]
}

add.alpha <- function (cols, alpha) rgb(t(col2rgb(cols)/255), alpha = alpha)

# all-cause life table

life.table <- function (mx, cohort = 100000, EX = T) { # if EX is true, just return life expectancy
  n <- length(mx) + 1
  qx <- 2 * mx / (2 + mx)
  qx <- c(qx, 1) # forced method - mortality rate max age + 1 is 100%
  lx <- c(1, cumprod(1 - qx)) * cohort
  dx <- -c(diff(lx), lx[n] * qx[n])
  t <- (lx + c(lx[-1], 0)) / 2
  Tx <- rev(cumsum(rev(t)))
  ex <- Tx / lx
  lt <- data.frame(lx = lx, dx = dx, t = t, Tx = Tx, ex = ex)[1:n,]
  if (EX) ex[1] else lt
}

# cause-specific life table function
# this works by creating a life table based on the all-cause mortality rates
# it then applies the proportions of deaths at each age
# the 'mat' argument is a matrix of cause-specific mortality rates

cs.life.table <- function (mat, cohort = 100000) {
  # all-cause life table
  mx <- rowSums(mat)
  n <- length(mx) + 1
  qx <- 2 * mx / (2 + mx)
  qx <- c(qx, 1) # forced method - mortality rate max age + 1 is 100%
```

```
  lx <- c(1, cumprod(1 - qx)) * cohort
  dx <- -c(diff(lx), lx[n] * qx[n])
  # make life table data frame
  lt <- data.frame(lx = lx, dx = dx)[1:n,]
  # matrix of proportions of deaths by cause at each age
  cm <- rbind(mat, 1) / rowSums(rbind(mat, 1))
  cs.dx <- lt$dx * cm
  colnames(cs.dx) <- paste0('dx_', colnames(mat))
  # outputs
  cbind(lt, cs.dx)[-nrow(lt),]
}

# ==============================
# read data and aggregate by sex
# ------------------------------

# data is from the 'HUPIO' study of people who use illicit opioids
# Codelist: https://wellcomeopenresearch.org/articles/5-282
# Causes of death: https://www.thelancet.com/journals/lanpub/article/PIIS2468-2667(21)00254-
1/fulltext

m <- fread("https://raw.githubusercontent.com/danlewer/hupio/main/mortality/single-year-of-age-
rates/hupio_rates_drugs_vs_smoking_25aug2023.csv", drop = 'gender', col.names = c('opioids', 'age',
'smoking', 'cvd', 'other_cancers', 'drug_related', 'hep', 'other', 'follow_up', 'all_cause'))
m <- m[opioids == T & age <= 70, lapply(.SD, sum), by = 'age'][, -'opioids']

# ===================================
# estimate age-specific mortality rates
# -----------------------------------

causes <- c('all_cause', 'smoking', 'drug_related', 'hep', 'cvd', 'other_cancers', 'other')
titles <- c('All cause', 'Smoking\n-specific\n(respiratory cancers\nand COPD)', 'Drug\npoisoning',
'Viral\nhepatitis', 'Cardiovascular\ndiseases', 'Other\ncancers\n(non-respiratory)',
'Other\nunderlying causes\nof death')

# estimate age-specific mortality rates using poisson model and cubic splines

pf <- function (outcome, nd = data.table(age = 18:70, follow_up = 1), ci = T, data = m) {
  f <- as.formula(paste0(outcome, '~ ns(age, 3) + offset(log(follow_up))'))
  model <- glm(f, data = data, family = 'poisson')
  inv <- model$family$linkinv
  p <- predict(model, newdata = nd, se.fit = T)
  if (ci == T) {
    cbind(nd,
          pred = inv(p$fit),
          lower = inv(p$fit - qnorm(0.975) * p$se.fit),
          upper = inv(p$fit + qnorm(0.975) * p$se.fit))
  } else {
    inv(p$fit)
  }
}
pm <- lapply(causes, pf)
names(pm) <- causes

# does the sum of modelled cause specific rates approximate the all-cause rate?

sum_cause_specific <- rowSums(sapply(pm[causes[-1]], function (x) x$pred))
plot(18:70, pm$all_cause$pred * 1e5, type = 'l', col = 'red', xlab = 'Age', ylab = 'Modelled
mortality rate / 100,000', main = 'All-cause (red) vs.\nsum of cause-specific (blue)')
lines(18:70, sum_cause_specific * 1e5, col = 'blue')

# visualise mortality rates by age with confidence intervals
```

```
cols <- brewer.pal(3, 'Set1')[3:1]

emf('FigA.emf', height = 6.5, width = 8, units = 'in')

par(mfrow = c(2, 3), mar = c(2, 3, 1, 1), oma = c(3, 3, 0, 0))
lapply(1:6, function (i) {
  y <- m[, get(causes[i])] / m$follow_up * 1e5
  ymax <- roundup(max(y), 2)
  plot(1, type = 'n', xlab = NA, ylab = NA, xlim = c(18, 70), ylim = c(0, ymax), axes = F)
  rect(18, 0, 70, ymax, col = 'grey98')
  points(18:70, y)
  axis(1, c(18, 3:7 * 10), pos = 0)
  axis(2, yax(ymax), pos = 18, las = 2)
  text(20, ymax * 0.98, titles[i], adj = c(0, 1))
  with(pm[causes[i]][[1]], {
    polygon(c(age, rev(age)), c(lower, rev(pmin(upper, ymax/1e5))) * 1e5, border = NA, col =
add.alpha(cols[1], 0.3))
    lines(18:70, pred * 1e5, col = cols[1])
  })
})
mtext('Age', side = 1, line = 1, outer = T, cex = 0.8)
mtext('Deaths per 100,000 person-years', side = 2, line = 1, outer = T, cex = 0.8)

dev.off()

# visualise mortality rates by age in single plot

causes2 <- causes[-c(1, 7)]
titles <- c('Respiratory cancers\nand COPD', 'Drug\npoisoning', 'Viral\nhepatitis',
'Cardiovascular\ndiseases', 'Cancers other\nthan repiratory')
cols <- brewer.pal(5, 'Set1')
pchs <- 0:4
final_vals <- sapply(pm[causes2], function (x) x$pred)[53,]

png('Fig1.png', height = 6, width = 9, units = 'in', res = 300)

par(mar = c(5, 5, 0, 13), xpd = NA)
plot(1, type = 'n', xlim = c(18, 70), ylim = c(0, 1300), axes = F, xlab = NA, ylab = 'Mortality rate
per 100,000 pys')
for (i in seq_along(causes2)) {
  y <- m[, get(causes2[i])] / m$follow_up * 100000
  y[y > 1300] <- NA
  points(m$age, y, col = cols[i], cex = 0.7, pch = pchs[i])
  y <- pm[[causes2[i]]]$pred * 100000
  lines(m$age, pmin(y, 1300), col = cols[i])
}
axis(1, 2:7 * 10, pos = 0)
axis(1, c(18, 70), labels = F, pos = 0)
axis(2, 0:13 * 100, pos = 18, las = 2)
rect(18, 0, 70, 1300)
title(xlab = 'Age', line = 2)
ys <- seq(400, 1200, length.out = 5)
segments(72, ys, 78, col = cols[order(final_vals)])
points(rep(75, 5), ys, pch = pchs[order(final_vals)], col = cols[order(final_vals)])
text(79, ys, titles[order(final_vals)], adj = 0)

dev.off()

# ============================================
# estimate deaths and YLLs in simulated datasets
# --------------------------------------------
```

```
# simulate deaths & YLLs in given scenario

deaths_ylls <- function (sims = 100,
                         smoking = 1, # eliminate smoking - set to 0
                         drugs = 1, # eliminate drugs - set to 0
                         point = F,
                         DATA = m,
                         fractions = cbind(smoking = c(1, 0), # first row is proportion attributable
to smoking, second to drugs
                                           cvd = c(0.5, 0),
                                           other_cancers = c(0.3, 0),
                                           drug_related = c(0, 1),
                                           hep = c(0, 1),
                                           other = c(0, 0)),
                         summary_only = T) {
  fractions <- fractions[,causes[-1]] # order fractions input correctly
  lapply (1 : if (point) 1 else sims, function(x) {
    if (x %% 100 == 0) print (x)
    m_sim <- sapply(DATA[, causes, with = F], function (x) rpois(length(x), x))
    m_sim <- cbind(DATA[, c('age', 'follow_up')], m_sim)
    mr <- as.data.frame(`names<-`(lapply(causes, pf, ci = F, data = if (point) DATA else m_sim),
causes)) # modelled rates
    smoking_rates <- t(t(mr[,-1]) * fractions[1,])
    colnames(smoking_rates) <- paste0('SMOKING', '_', causes[-1])
    drug_rates <- t(t(mr[,-1]) * fractions[2,])
    colnames(drug_rates) <- paste0('DRUGS', '_', causes[-1])
    other_rates <- mr[,-1] - (smoking_rates + drug_rates)
    smoking_rates <- smoking_rates * smoking
    drug_rates <- drug_rates * drugs
    colnames(other_rates) <- paste0('OTHER', '_', causes[-1])
    mat <- cbind(drug_rates, smoking_rates, other_rates)
    cs.lt <- cs.life.table(mat)
    r <- list(life_table = cs.lt,
              summary = rbind(deaths = colSums(cs.lt[-53,])[-(1:2)],
                              ylls = colSums(cs.lt[-53,] * (69 - 18:69 + 0.5))[-(1:2)]))
    if (summary_only) r$summary else r
  })
}

# generate results in different scenarios of smoking and drugs

set.seed(562)
B <- 1000 # number of sims

Ap <- deaths_ylls(point = T, smoking = 1, drugs = 1)
Am <- deaths_ylls(sims = B, smoking = 1, drugs = 1)
Bp <- deaths_ylls(point = T, smoking = 1, drugs = 0)
Bm <- deaths_ylls(sims = B, smoking = 1, drugs = 0)
Cp <- deaths_ylls(point = T, smoking = 0, drugs = 1)
Cm <- deaths_ylls(sims = B, smoking = 0, drugs = 1)
Dp <- deaths_ylls(point = T, smoking = 0, drugs = 0)
Dm <- deaths_ylls(sims = B, smoking = 0, drugs = 0)

# function to summarise results of simulations

smoking_vars <- paste0('dx_', 'SMOKING', '_', causes[-1])
drug_vars <- paste0('dx_', 'DRUGS', '_', causes[-1])
s <- function (d, i = 1) { # 1 = deaths, 2 = YLLS
  if (length(d) == 1) {
    x <- d[[1]][i,]
    x <- c(all = sum(x), smoking = sum(x[smoking_vars]), drugs = sum(x[drug_vars]))
```

```
    x <- c(x, other = x[1] - (x[2] + x[3]))
    return(c(x, pc_smoking = x[2] / x[1] * 100, pc_drugs = x[3] / x[1] * 100, pc_other = x[4] / x[1]
* 100))
  }
  x <- t(sapply(d, function (x) x[i,]))
  x <- cbind(all = rowSums(x), smoking = rowSums(x[, smoking_vars]), drugs = rowSums(x[, drug_vars]))
  x <- cbind(x, other = x[,1] - (x[,2] + x[,3]))
  cbind(x, pc_smoking = x[,2] / x[,1] * 100, pc_drugs = x[,3] / x[,1] * 100, pc_other = x[,4] / x[,1]
* 100)
}

# create deaths results

f <- function (x, digs = 1, units = c(1000, 1000, 1000, 1000, 1, 1, 1)) {
  x <- t(t(x) / units)
  x <- formatC(round(x, digs), big.mark = ',', format = 'f', digits = digs)
  `names<-`(paste0(x[1,], ' (', x[3,], ', ', x[4,], ')'), colnames(x))
}
deaths_scenarios <- rbind(cbind(observed = f(rbind(point = s(Ap), apply(s(Am), 2, quantile, probs =
c(0.5, 0.025, 0.975)))),
                                eliminate_drugs = f(rbind(point = s(Bp), apply(s(Bm), 2, quantile,
probs = c(0.5, 0.025, 0.975)))),
                                eliminate_smoking = f(rbind(point = s(Cp), apply(s(Cm), 2, quantile,
probs = c(0.5, 0.025, 0.975)))),
                                eliminate_both = f(rbind(point = s(Dp), apply(s(Dm), 2, quantile,
probs = c(0.5, 0.025, 0.975))))),
                          cbind(observed = NA,
                                eliminate_drugs = f(rbind(point = s(Bp) - s(Ap), apply(s(Bm) - s(Am),
2, quantile, probs = c(0.5, 0.025, 0.975)))),
                                eliminate_smoking = f(rbind(point = s(Cp) - s(Ap), apply(s(Cm) -
s(Am), 2, quantile, probs = c(0.5, 0.025, 0.975)))),
                                eliminate_both = f(rbind(point = s(Dp) - s(Ap), apply(s(Dm) - s(Am),
2, quantile, probs = c(0.5, 0.025, 0.975)))))

# create YLL results

f2 <- function (x) f(x, digs = 2, units = c(100000, 100000, 100000, 100000, 1, 1, 1))
yll_scenarios <- rbind(cbind(observed = f2(rbind(point = s(Ap, 2), apply(s(Am, 2), 2, quantile, probs
= c(0.5, 0.025, 0.975)))),
                             eliminate_drugs = f2(rbind(point = s(Bp, 2), apply(s(Bm, 2), 2,
quantile, probs = c(0.5, 0.025, 0.975)))),
                             eliminate_smoking = f2(rbind(point = s(Cp, 2), apply(s(Cm, 2), 2,
quantile, probs = c(0.5, 0.025, 0.975)))),
                             eliminate_both = f2(rbind(point = s(Dp, 2), apply(s(Dm, 2), 2, quantile,
probs = c(0.5, 0.025, 0.975))))),
                       cbind(observed = NA,
                             eliminate_drugs = f2(rbind(point = s(Bp, 2) - s(Ap, 2), apply(s(Bm, 2) -
s(Am, 2), 2, quantile, probs = c(0.5, 0.025, 0.975)))),
                             eliminate_smoking = f2(rbind(point = s(Cp, 2) - s(Ap, 2), apply(s(Cm, 2)
- s(Am, 2), 2, quantile, probs = c(0.5, 0.025, 0.975)))),
                             eliminate_both = f2(rbind(point = s(Dp, 2) - s(Ap, 2), apply(s(Dm, 2) -
s(Am, 2), 2, quantile, probs = c(0.5, 0.025, 0.975)))))

# save table

scenarios <- rbind(deaths_scenarios, yll_scenarios)
write.csv(scenarios, 'scenarios_table.csv')

# ========================
# plot of cumulative deaths
# ------------------------
```

```
cuml_deaths <- deaths_ylls(point = T, summary_only = F)[[1]]$life_table
cuml_deaths <- sapply(cuml_deaths, cumsum)[, -(1:2)]

labs <- outer(c('dx_DRUGS_', 'dx_SMOKING_', 'dx_OTHER_'), causes[-1], paste0)
cols <- brewer.pal(6, 'Set3')

ynums <- apply(cuml_deaths, 1, cumsum)
ynums <- rbind(0, ynums)
ynums2 <- ynums[c(1, 7, 13, 19),]

sdo70 <- tapply(cuml_deaths[53,], rep(1:3, each = 6), sum)
sdo69 <- tapply(cuml_deaths[52,], rep(1:3, each = 6), sum)
labs3 <- c('Attributable to illict drugs', 'Attributable to tobacco', 'Attributable to other
factors')
labs3 <- paste0(labs3,
                "\n", paste0(round(sdo69 / 1000, 1), '%', ' cumulative risk'),
                "\n", paste0(round(sdo69 / sum(sdo69) * 100, 1), '%', ' of premature deaths'))

png('Fig2.png', height = 7, width = 10, res = 300, units = 'in')
par(mar = c(5, 6, 0, 15), xpd = NA)
plot(1, type = 'n', xlim = c(18, 70), ylim = c(0, 100000), xlab = NA, ylab = NA, axes = F)
for (i in 1:ncol(cuml_deaths)) {
  polygon(c(18:70, 70:18), y = c(ynums[i,], rev(ynums[i+1,])), col = rep(cols, 3)[i], border =
'white')
}
for (i in 1:3) {
  polygon(c(18:70, 70:18), y = c(ynums2[i,], rev(ynums2[i+1,])), border = 'black', lwd = 1.5)
}
rect(18, 0, 70, 100000, lwd = 1.5)
axis(1, pos = 0)
axis(2, 0:5 * 20000, paste0(0:5 * 20, '%'), las = 2, pos = 18)
title(xlab = 'Age', line = 2)
title(ylab = 'Cumulative risk of death in a cohort of\npeople who use illicit opioids', line = 4)
ys <- seq(20000, 97500, length.out = 7)
rect(20, ys[-length(ys)], 23, ys[-1], col = cols)
text(25, ys[-length(ys)] + diff(ys)/2, c('Respiratory cancers\nand COPD', 'Drug\npoisoning',
'Viral\nhepatitis', 'Cardiovascular\ndiseases', 'Cancers other\nthan respiratory', 'All
other\ndeaths'), adj = 0)
arrows(73, c(0, cumsum(sdo70)[-3]) + 500, y1 = cumsum(sdo70) - 500, code = 3, length = 0.1, angle =
45)
text(75, c(0, cumsum(sdo70))[-4] + diff(c(0, cumsum(sdo70)))/2, labs3, adj = 0)
dev.off()

# =============================================
# bar plot of attributable and prevented deaths
# ---------------------------------------------

x <- rbind(s(Ap), s(Bp), s(Cp), s(Dp)) / 1e5
y1 <- t(apply(cbind(0, x[,-1]), 1, cumsum))
xl <- 0:3 * 4
delta <- -t(x[1,] - t(x))
waterfall_targets <-t(apply(delta[-1,-1], 1, function (y) x[1,1] + cumsum(y)))

# waterfall chart

waterfall <- function (start, targets, xleft, width = 0.5, cols = 1:3) {
  yvals <- cbind(c(0, start, targets), c(start, targets, 0))[2:(length(targets)+1),]
  mapply(rect,
         xleft = xleft,
         ybottom = apply(yvals, 1, min),
         xright = xleft + width,
         ytop = apply(yvals, 1, max),
```

```
         col = cols)
  segments(x0 = xleft, y0 = targets, x1 = xleft + width * 2, lty = 3)
}

cols <- brewer.pal(3, 'Set2')
ys <- seq(0.4, 0.7, length.out = 4)

png('Fig3.png', height = 5, width = 8, units = 'in', res = 300)
par(mar = c(6, 5, 0, 10), xpd = NA)
plot(1, type = 'n', xlim = c(0, 12.75), ylim = c(0, 0.7), axes = F, xlab = NA, ylab = NA)
rect(-0.25, 0:6/10, 12.75, 1:7/10, col = rep(c('white', 'grey93'), 4), border = NA)
rect(-0.25, 0, 12.75, 0.7)
segments(-0.25, x[1,1], x1 = 12.75, lty = 3)
mapply(rect,
       xleft = xl,
       ybottom = split(y1[,-4], f = 1:4),
       xright = xl + 0.5,
       ytop = split(y1[,-1], f = 1:4),
       col = rep(list(cols), each = 4))
waterfall(start = x[1,1], targets = waterfall_targets[1,], xleft = 1:3, cols = cols)
waterfall(start = x[1,1], targets = waterfall_targets[2,], xleft = 5:7, cols = cols)
waterfall(start = x[1,1], targets = waterfall_targets[3,], xleft = 9:11, cols = cols)
segments(c(-0.25, 0.75, 4.75, 8.75, 12.75), y0 = 0, y1 = 0.7)
axis(2, 0:7 / 10, paste0(0:7 * 10, '%'), las = 2, pos = -0.25)
title(ylab = 'Risk of death before age 70')
text(0.25, -0.01, 'Observed\nmortality rates', srt = 90, adj = 1)
text(c(2.75, 6.75, 10.75), -0.01, c('Eliminating\nillicit\ndrugs', 'Eliminating\ntobacco\nsmoking',
'Eliminating illicit\ndrugs and\ntobacco\nsmoking'), adj = c(0.5,1))
rect(13, ys[-length(ys)], 13.5, ys[-1], col = cols)
text(13.75, ys[-length(ys)] + diff(ys)/2, c('Attributable to\nillicit drugs', 'Attributable
to\ntobacco smoking', 'Attributable to\nother factors'), adj = 0)
dev.off()

# ==================================
# comparison with general population
# ----------------------------------

# gen pop data

mgp <- fread("https://raw.githubusercontent.com/danlewer/hupio/main/mortality/single-year-of-age-
rates/hupio_rates_drugs_vs_smoking_25aug2023.csv", drop = 'gender', col.names = c('opioids', 'age',
'smoking', 'cvd', 'other_cancers', 'drug_related', 'hep', 'other', 'follow_up', 'all_cause'))
mgp <- mgp[opioids == F & age <= 70, lapply(.SD, sum), by = 'age'][, -'opioids']
s(deaths_ylls(point = T, DATA = mgp))
s(deaths_ylls(point = T, DATA = mgp), i = 2) / 100000

# ==============================
# stimulant sensitivity analysis
# ------------------------------

ss <- function (x,
                f = cbind(smoking = c(1, 0), # first row is proportion attributable to smoking,
second to drugs
                          cvd = c(0.5, 0),
                          other_cancers = c(0.3, 0),
                          drug_related = c(0, 1),
                          hep = c(0, 1),
                          other = c(0, 0)),
                lt = F) {
  f[2,2] <- x
  if (lt) return (deaths_ylls(point = T, fractions = f, summary_only = F)[[1]]$life_table)
  s(deaths_ylls(point = T, fractions = f))
```

```
}
vals <-seq(0, 0.5, 0.05)
sens <- t(sapply(vals, ss))
sens[,1:4] <- sens[,1:4] / 1e3
sens <- round(sens, 1)
sens <- cbind(att = vals * 100, sens)
write.csv(sens, 'sens.csv')

# plot where PAF = 0.5

cuml_deaths <- ss(0.3, lt = T)
cuml_deaths <- sapply(cuml_deaths, cumsum)[, -(1:2)]

cols <- brewer.pal(6, 'Set3')

ynums <- apply(cuml_deaths, 1, cumsum)
ynums <- rbind(0, ynums)
ynums2 <- ynums[c(1, 7, 13, 19),]

sdo70 <- tapply(cuml_deaths[53,], rep(1:3, each = 6), sum)
sdo69 <- tapply(cuml_deaths[52,], rep(1:3, each = 6), sum)
labs3 <- c('Attributable to illict drugs', 'Attributable to tobacco', 'Attributable to other
factors')
labs3 <- paste0(labs3,
                "\n", paste0(round(sdo69 / 1000, 1), '%', ' cumulative risk'),
                "\n", paste0(round(sdo69 / sum(sdo69) * 100, 1), '%', ' of premature deaths'))

emf('FigS2.emf', height = 7, width = 10)
par(mar = c(5, 6, 0, 15), xpd = NA)
plot(1, type = 'n', xlim = c(18, 70), ylim = c(0, 100000), xlab = NA, ylab = NA, axes = F)
for (i in 1:ncol(cuml_deaths)) {
  polygon(c(18:70, 70:18), y = c(ynums[i,], rev(ynums[i+1,])), col = rep(cols, 3)[i], border =
'white')
}
for (i in 1:3) {
  polygon(c(18:70, 70:18), y = c(ynums2[i,], rev(ynums2[i+1,])), border = 'black', lwd = 1.5)
}
rect(18, 0, 70, 100000, lwd = 1.5)
axis(1, pos = 0)
axis(2, 0:5 * 20000, paste0(0:5 * 20, '%'), las = 2, pos = 18)
title(xlab = 'Age', line = 2)
title(ylab = 'Cumulative risk of death in a cohort of\npeople who use illicit opioids', line = 4)
ys <- seq(20000, 97500, length.out = 7)
rect(20, ys[-length(ys)], 23, ys[-1], col = cols)
text(25, ys[-length(ys)] + diff(ys)/2, c('Respiratory cancers\nand COPD', 'Drug\npoisoning',
'Viral\nhepatitis', 'Cardiovascular\ndiseases', 'Cancers other\nthan respiratory', 'All
other\ndeaths'), adj = 0)
arrows(73, c(0, cumsum(sdo70)[-3]) + 500, y1 = cumsum(sdo70) - 500, code = 3, length = 0.1, angle =
45)
text(75, c(0, cumsum(sdo70))[-4] + diff(c(0, cumsum(sdo70)))/2, labs3, adj = 0)
dev.off()
```

8. References

1. Walter SD. Calculation of Attributable Risks from Epidemiological Data. Int J Epidemiol. 1978;7: 175–182. doi:10.1093/ije/7.2.175

2. Lewer D, Brothers TD, Van Hest N, Hickman M, Holland A, Padmanathan P, et al. Causes of death among people who used illicit opioids in England, 2001–18: a matched cohort study. The Lancet Public Health. 2022;7: e126–e135. doi:10.1016/S2468-2667(21)00254-1

3. Doll R, Peto R, Boreham J, Sutherland I. Mortality in relation to smoking: 50 years' observations on male British doctors. BMJ. 2004;328: 1519. doi:10.1136/bmj.38142.554479.AE

4. Ordóñez-Mena JM, Schöttker B, Mons U, Jenab M, Freisling H, Bueno-de-Mesquita B, et al. Quantification of the smoking-associated cancer risk with rate advancement periods: meta-analysis of individual participant data from cohorts of the CHANCES consortium. BMC Med. 2016;14: 62. doi:10.1186/s12916-016-0607-5

5. Kaye C, Darke S, Topp L. An examination of cocaine dependence among injecting and non-injecting drug users in Sydney. Sydney; 2001. Report No.: 116. Available: https://www.unsw.edu.au/research/ndarc/resources/an-examination-of-cocaine-dependence-among-injecting-and-non-inj

6. Degenhardt L, Peacock A, Colledge S, Leung J, Grebely J, Vickerman P, et al. Global prevalence of injecting drug use and sociodemographic characteristics and prevalence of HIV, HBV, and HCV in people who inject drugs: a multistage systematic review. The Lancet Global Health. 2017;5: e1192–e1207. doi:10.1016/S2214-109X(17)30375-3

7. UK Health Security Agency. Unlinked Anonymous Monitoring (UAM) Survey of HIV and viral hepatitis among people who inject drugs (PWID): 2024 report. UK Government; 2024. Available: https://www.gov.uk/government/publications/people-who-inject-drugs-hiv-and-viral-hepatitis-monitoring/unlinked-anonymous-monitoring-uam-survey-of-hiv-and-viral-hepatitis-among-people-who-inject-drugs-pwid-2024-report

8. Office for Health Improvement and Disparitie, UK Health Security Agency. Estimates of opiate and crack use in England: main points and methods. UK Government; 2023. Available: https://www.gov.uk/government/publications/opiate-and-crack-cocaine-use-prevalence-estimates/estimates-of-opiate-and-crack-use-in-england-main-points-and-methods

9. Gan WQ, Buxton JA, Scheuermeyer FX, Palis H, Zhao B, Desai R, et al. Risk of cardiovascular diseases in relation to substance use disorders. Drug and Alcohol Dependence. 2021;229: 109132. doi:10.1016/j.drugalcdep.2021.109132

10. Talarico GP, Crosta ML, Giannico MB, Summaria F, Calò L, Patrizi R. Cocaine and coronary artery diseases: a systematic review of the literature. Journal of Cardiovascular Medicine. 2017;18: 291–294. doi:10.2459/JCM.0000000000000511

11. Sordo L, Indave BI, Barrio G, Degenhardt L, de la Fuente L, Bravo MJ. Cocaine use and risk of stroke: A systematic review. Drug and Alcohol Dependence. 2014;142: 1–13. doi:10.1016/j.drugalcdep.2014.06.041

12. Arenas DJ, Beltran S, Zhou S, Goldberg LR. Cocaine, cardiomyopathy, and heart failure: a systematic review and meta-analysis. Sci Rep. 2020;10: 19795. doi:10.1038/s41598-020-76273-1

13. Colell E, Domingo-Salvany A, Espelt A, Parés-Badell O, Brugal MT. Differences in mortality in a cohort of cocaine use disorder patients with concurrent alcohol or opiates disorder. Addiction. 2018;113: 1045–1055. doi:10.1111/add.14165
